# Astragaloside IV Exerts a Myocardial Protective Effect against Cardiac Hypertrophy in Rats, Partially via Activating the Nrf2/HO-1 Signaling Pathway

**DOI:** 10.1155/2019/4625912

**Published:** 2019-06-11

**Authors:** Pei Nie, Fanjing Meng, Jinguo Zhang, Xiqing Wei, Cheng Shen

**Affiliations:** ^1^Jining Medical University, Jining 272067, China; ^2^Department of Cardiology, Affiliated Hospital of Jining Medical University, Jining 272029, China

## Abstract

Previous evidence suggested that astragaloside IV (ASIV) had a cardioprotective effect, but the potential mechanisms were undetermined. This study is aimed at validating the prevention of cardiac hypertrophy in chronic heart failure (CHF) rats and hypertrophy in H9c2 cardiomyocytes by ASIV and at exploring the potential mechanism involved. CHF rat models of abdominal aortic constriction (AAC) were used with the aim of determining the protective effect of ASIV in cardiac hypertrophy in the rats. We proved that ASIV could attenuate cardiac hypertrophy by improving left ventricular function and structure and showed that the expression of nuclear factor-erythroid 2-related factor 2 (Nrf2) and its downstream gene heme oxygenase-1 (HO-1) increased in the high-dose ASIV intervention group. To further investigate the specific mechanism of ASIV, we hypothesized that ASIV might prevent cardiac hypertrophy via activating the Nrf2/HO-1 signaling pathway. We established a cardiomyocyte hypertrophy model induced by angiotensin II (Ang II), which was then transfected with Nrf2 shRNA, to knock down the expression of the Nrf2 gene. We found that the protective effect of ASIV against Ang II-induced cardiomyocyte hypertrophy was abolished in the Nrf2 shRNA transfection group, ultimately aggravating cardiomyocyte hypertrophy induced by Ang II, and it is possible that oxidative stress may be involved in this process. Our results demonstrated that ASIV improved cardiac function and inhibited cardiac hypertrophy by upregulating Nrf2, and this effect was partially achieved by stimulating the Nrf2/HO-1 signaling pathway, suggesting that ASIV could have therapeutic potential for the treatment of cardiac hypertrophy and CHF.

## 1. Introduction

Chronic heart failure (CHF) is caused by compensatory changes in the heart induced by long-term insufficient cardiac output [1, 2]. Epidemiological data show that the number of CHF patients increases each year as the population ages [3, 4]. Previous studies showed that the estimated 5-year survival rate of CHF patients is 50%, while the 10-year survival rate is estimated to be 10%; these rates are even lower in developing countries [5, 6]. Cardiac hypertrophy, which is characterized by a substantial increase in cardiomyocyte size, is divided into two categories: physiological hypertrophy and pathological hypertrophy [7–9]. Physiological hypertrophy is benign, compensatory, and adaptive, whereas pathological hypertrophy is deleterious and can lead to decompensation, diastolic dysfunction, and ultimately CHF and sudden death [10–12]. It has been reported that sustained pathological cardiac hypertrophy is a critical factor in the progression of CHF and can serve as a predictor of CHF development [13]. Therefore, preventative strategies against the incidence of cardiac hypertrophy may be important for inhibiting the development of CHF and improving outcomes in CHF patients.

Astragaloside IV (ASIV) is an active component of *Astragalus membranaceus*, a traditional Chinese medicine, and has many pharmacological functions, such as potent anti-inflammatory, antifibrotic, antioxidant, antiasthma, and immune-regulatory effects [14]. Numerous studies have shown that the administration of ASIV may facilitate the alleviation of cardiovascular diseases such as hypertension, myocardial infarction, and cardiomyopathy [15–17]. Moreover, the specific protective effects of ASIV may oppose processes such as cardiomyocyte hypertrophy, apoptosis, and fibrosis [18–22]. Some studies have suggested that the protective effect of ASIV against cardiac hypertrophy is related to the regulation of cardiac-related signaling pathways, such as inhibition of the TLR4/NF-*κ*B signaling pathway and the Ca^2+^/CaN signaling pathway and regulation of NF-*κ*B/PGC-1*α* signaling-mediated energy biosynthesis [18, 19, 23, 24]. Other studies have shown that ASIV can protect brain tissue by reducing oxidative stress and mediating related signaling pathways [25]. However, there is no potent evidence demonstrating the phenomenon involved in the antihypertrophic effect of ASIV.

Transcription factor nuclear factor-erythroid 2-related factor 2 (Nrf2, also known as NFE2L2) is a key regulator of redox homeostasis and cellular antioxidant defenses. Under homeostatic conditions, Nrf2 is sequestered in the cytoplasm by Kelch-like ECH-associated protein 1 (Keap1), which is a homodimeric protein that bridges Nrf2 with the E3 ligase complex [26, 27]. When exposed to external stimulation, the oxidative stress system is activated, and Nrf2 dissociates from Keap1 and the E3 ligase complex, then translocates to the nucleus [28, 29]. Previous studies revealed a discrete regulatory element in the promoter region of genes encoding phase II drug metabolism enzymes; this cis-regulatory element is referred to as antioxidant response element (ARE) and is involved in the progression of Nrf2 transcription factor-regulated genes [30]. Nrf2 can activate antioxidant defense enzymes including HO-1 and NAD(P)H dehydrogenase quinone 1 (NQO1) and thereby exert an antioxidant effect [31]. Our previous studies showed that activation of Nrf2 protects the heart against ischemia-reperfusion injury, diabetic cardiomyopathy, and angiotensin II- (Ang II-) induced cardiomyopathy [32–35]. Other studies have shown that activation of Nrf2 is associated with the suppression of Ang II-induced oxidative stress in cardiomyocytes [36].

Currently, it is not known whether ASIV can protect the heart through antioxidant stress or what specific mechanism may be involved. Therefore, the present study was aimed at determining whether ASIV exerts cardiovascular protective effects in CHF rat models of abdominal aortic constriction (AAC) and hypertrophic cardiomyocytes induced by Ang II and at dissecting the underlying mechanisms connecting the activation of the Nrf2/HO-1 pathway.

## 2. Materials and Methods

### 2.1. Antibodies and Reagents

ASIV was purchased from Nanjing Spring and Autumn Biological Engineering (Nanjing, China). Benazepril HCL was purchased from the Novartis Pharmaceutical Corporation (Beijing, China). Angiotensin II (Ang II) was obtained from the Shanghai YuanYe Biotechnology Co. Ltd. (Shanghai, China). Polyclonal antibodies against *α*-actinin, Nrf2, and *β*-actin and secondary antibodies were purchased from ABclonal (Wuhan, China). Monoclonal antibodies against atrial natriuretic peptide (ANP), brain natriuretic titanium (BNP), and HO-1 were purchased from Abcam (USA). The total RNA extraction kit and cDNA synthesis kit were purchased from Vazyme Biotechnology (Nanjing, China). The primers for ANP, Nrf2, HO-1, and glyceraldehyde phosphate dehydrogenase (GAPDH) were designed and synthesized by Dingguo Biotechnology (Beijing, China). The BNP enzyme-linked immunosorbent assay (ELISA) kit and dimethyl sulfoxide (DMSO) were purchased from Apuris Biological Technology (Beijing, China). EP50 tubes were purchased from BDTar BioTech (Shanghai, China). The bicinchoninic acid (BCA) protein assay kit and enhanced chemiluminescence kit were purchased from Beyotime Biotechnology (Haimen, China).

### 2.2. Animals and Treatment

Fifty adult rats (Experimental Animal Centre, Shandong, China; certificate number: SCXK20140007) of SPF grade weighing 240 ± 10 g were housed in a controlled environment (23 ± 2°C, 45% ± 5% humidity, and a 12 h dark/12 h light cycle). The experiments were conducted according to the principles approved by the Animal Care and Use Committee of the Affiliated Hospital of Jining Medical University.

### 2.3. Establishment and Evaluation of CHF Model Rats and Drug Treatment

Rats were divided into 5 groups (*N* = 10 per group) in accordance with a random number table. The groups included a sham operation group, a CHF group, a CHF+low-dose ASIV group, a CHF+high-dose ASIV group, and a CHF+benazepril HCL group. CHF was induced by AAC as described previously [37]. The results of model establishment were evaluated by ultrasonic cardiogram (UCG) at 8 weeks after the surgery. All animals received drug/placebo treatment for 8 weeks after we confirmed that the CHF model was successfully established. The cardiac function indexes of the 5 groups were evaluated with UCG. The rats in the CHF+low-dose and high-dose ASIV groups were administered with ASIV through intragastric gavage at 40 and 80 mg·kg^−1^·d^−1^, respectively, which was dissolved in 1 ml of 1% sodium dimethyl sulfoxide (DMSO). Rats in the CHF+benazepril HCL group were administered with the drug through intragastric gavage at 10 mg·kg^−1^·d^−1^, dissolved in 1 ml of 1% DMSO. Rats in the sham operation and CHF groups were administered with 1% DMSO at 1 ml/d through intragastric gavage as a placebo control.

### 2.4. Posttreatment Examination of Cardiac Structure and Function

After 8 weeks of treatment, all 5 groups were anesthetized with 3% isoflurane; the cardiac structural and functional parameters of all groups, including the left ventricular end-diastolic dimension (LVEDD), left ventricular end-systolic dimension (LVESD), left ventricular posterior wall depth (LVPWD), left ventricular ejection fraction (LVEF), left ventricular fractional shortening (LVFS), and early diastolic filling and atrial filling velocity ratio of the mitral flow (E/A) were measured noninvasively by echocardiography imaging using a Vevo 2100 high-resolution imaging system equipped with a transducer with center frequencies ranging from 13 to 24 MHz (MS250; VisualSonics, Toronto, 8 Canada).

### 2.5. Detection of Serum BNP Concentrations and Ventricular Hypertrophy Parameters

After UCG, the rats were sacrificed via intraperitoneal injection of chloral hydrate (4 mg/kg), and blood samples were collected for determination of the BNP concentration in serum. Their hearts were removed, and the heart-to-body weight (HW : BW) and heart weight-to-tibia length (HW : TL) ratios were used as a measure of cardiac hypertrophy. Part of the left ventricle was preserved in an -80°C freezer for Western blot and real-time quantitative polymerase chain reaction (RT-qPCR) analyses.

### 2.6. Cell Culture

Embryonic rat heart-derived cells (H9c2 cells) were purchased from the Beijing Institutes for Biological Sciences (Beijing, China) and maintained in a high-glucose Dulbecco's modified Eagle's medium (4.5 g/l glucose) supplemented with 10% fetal bovine serum (FBS) and antibiotics (50 U/ml penicillin and 50 *μ*g/ml streptomycin) in a humidified atmosphere of 5% CO_2_ and 95% O_2_ at 37°C. After culture for 24 h, H9c2 cardiomyocytes were stimulated with Ang II (1 *μ*M) for 24 h to induce a cardiomyocyte hypertrophy model. H9c2 cardiomyocytes were pretreated with astragaloside IV (ASIV) (25, 50, or 100 *μ*M) or full ASIV (100 *μ*M) for 1 h before Ang II administration. Ang II was dissolved in phosphate-buffered saline (PBS), and ASIV was dissolved in DMSO, where the final concentration of DMSO was less than 0.1%.

### 2.7. Cell Viability Assay

H9c2 cells were seeded into 96-well plates at 4 × 10^3^ cells/well and incubated for 12 h. To determine the optimal concentration and time of Ang II treatment, cells were administered with different concentration of Ang II and cultured for 12, 24, or 48 h. To test the cell viability under ASIV treatment, cells were administered with different concentrations of ASIV and cultured for 24 h. To evaluate cell viability under Ang II and ASIV treatment, cells were administered with Ang II, ASIV, or a combination of the two drugs. Next, the number of viable cells was evaluated by the CCK8 assay. Briefly, the cells were incubated with CCK8 at a final concentration of 0.1 mg/ml at the end of drug treatment. After 2 h, the number of viable cells was measured by evaluating the absorbance at 450 nm and 630 nm with a microplate reader.

### 2.8. Nrf2 shRNA Transfection

For Nrf2-shRNA transfection, H9c2 cells were seeded in 6-well plates at a density of 8 × 10^5^ cells/well and cultured for 16 h. The cells were then transfected with Nrf2-shRNA (1, 2, and 3) or Nrf2-negative control shRNA (Dingguo, Beijing, China) using the Lipofectamine®2000 reagent (Thermo, Carlsbad, CA) for 24 h. The transfected cells were treated with Ang II (1 *μ*M) alone, ASIV (100 *μ*M) alone, or Ang II (1 *μ*M)/ASIV (100 *μ*M) for 24 h. Consequently, protein and RNA samples from the cells were extracted and stored at -80°C for further analysis.

### 2.9. RT-qPCR Analysis

Total RNA was extracted from heart tissue and adherent H9c2 cardiomyocytes using a total RNA extraction kit according to the manufacturer's instructions, and RNA concentrations were detected over time. Total RNA was reverse transcribed into cDNA using a cDNA synthesis kit and then added to 20 *μ*l reaction mixtures that contained SYBR green, RNase-free water, a forward primer, a reverse primer, and cDNA to detect the relative mRNA expression of genes. The PCR program was as follows: 95°C for 3 min, followed by 40 cycles at 95°C for 10 s and 60°C for 30 s. Melting curves were used to ensure that only the correct product was amplified. The fold change difference compared to the control group was calculated using the 2^-ΔΔCt^ method.

### 2.10. Western Blot Analysis

Heart tissue and adherent H9c2 cardiomyocytes were lysed in RIPA buffer containing a mixture of protease inhibitors to extract proteins, and the protein concentration was determined with a BCA protein assay kit. Thirty microgram protein samples were electrophoresed and separated via sodium dodecyl sulfate polyacrylamide gel electrophoresis (SDS-PAGE), then transferred to polyvinylidene fluoride (PVDF) membranes. The membranes were subsequently incubated with primary antibodies and then with the corresponding secondary antibodies and were finally detected with an enhanced chemiluminescence kit and a gel imaging system (Shanghai Furi Co., Shanghai, China) [37].

### 2.11. Immunofluorescence Analysis

The cell surface area of H9c2 cells was assessed by immunofluorescent staining. The cells which were treated with different drugs were subsequently fixed with 4% polyoxymethylene, permeabilized with 0.1% Triton X-100 in PBS for 15 min and stained with *α*-actinin (1 : 100 dilution), followed by a fluorescent secondary antibody, after which the samples were analyzed by laser-scanning confocal microscopy (ZEISS LSM800, Shanghai, China) at 400x magnification. Surface areas were measured using ImageJ software.

### 2.12. Analysis of Reactive Oxygen Species (ROS) Levels

ROS were detected with 2,7-dichlorodihydrofluorescein diacetate (DCFH-DA). After removing the medium, the cells were incubated with DCFH-DA (10.0 *μ*M) for 20 min at 37°C, and the samples were then analyzed by laser-scanning confocal microscopy at 200x magnification.

### 2.13. Statistical Analysis

Quantification was performed by blinded researchers, while the other experiments were performed in a nonblinded manner. The data are expressed as the mean ± standard deviation (SD). Statistical analysis was performed with GraphPad Prism 5.0 software (San Diego, CA, USA) via one-way analysis of variance (ANOVA) followed by Dunnett's test when comparing multiple groups, whereas an unpaired *t*-test was applied when comparing two different groups. Statistical significance was considered at *P* < 0.05.

## 3. Results

### 3.1. Improvement of Cardiac Function after ASIV Intervention

**Figure 1:**
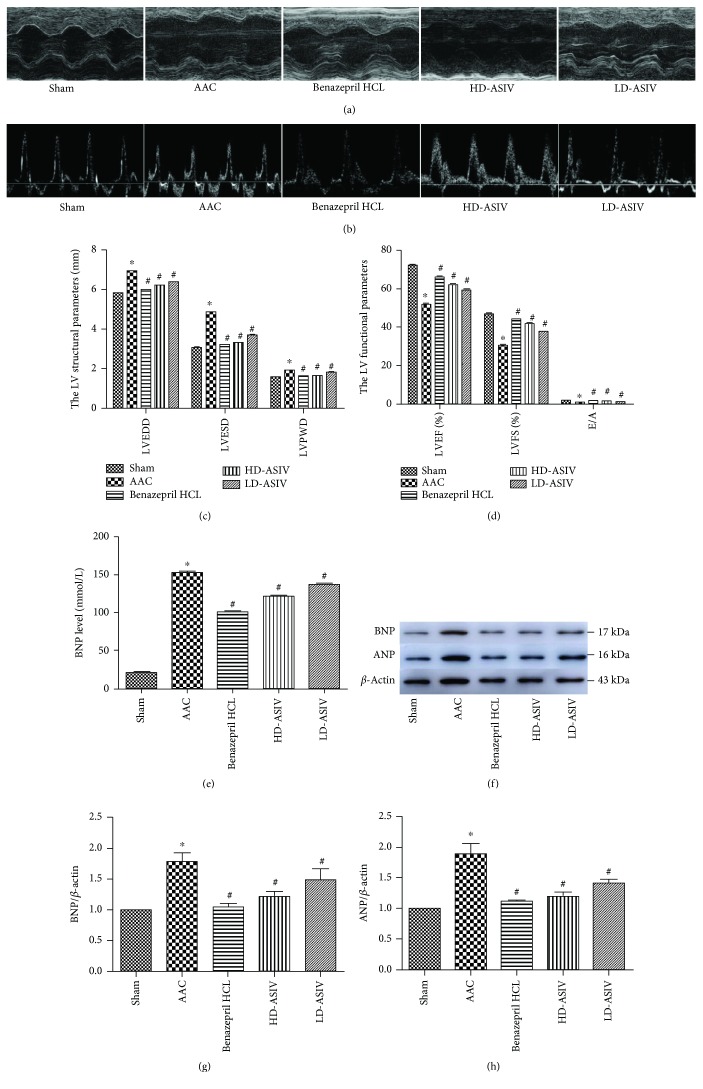
Cardiac function in the 5 experimental groups after intervention for 8 weeks. (a, b) Representative M-mode LV echocardiographs and mitral velocity profiles of the 5 experimental groups after intervention (*n* = 10 for the 5 experimental groups). (c, d) Quantitative data are presented as the means ± SD. ^∗^*P* < 0.05 vs. the sham operation group; ^#^*P* < 0.05 vs. the AAC group. (e) Serum BNP concentrations of the 5 experimental groups. Quantitative data are shown as the means ± SD (*n* = 10 for the 5 experimental groups). (f–h) Representative blots of BNP and ANP, and the intensity normalized against GAPDH, in the LV myocardium in the 5 groups (*n* = 10 for the 5 experimental groups). Quantitative data are presented as the means ± SD. ^∗^*P* < 0.05 vs. the sham operation group, ^#^*P* < 0.05 vs. the AAC group.

UCG was used to evaluate the effects of ASIV on cardiac structure and function in the 5 groups of rats after 8 weeks of intervention. From the aspect of cardiac function, the ASIV-treated rats showed improvements in the left ventricular function parameters compared to the CHF group, including increases in LVEF, LVFS, and E/A. Regarding cardiac structure, the ASIV-treated rats showed decreases in LVEDD, LVESD, and LVPWD compared to the CHF group (Figures 1(a)–1(d)). This basically satisfied the experimental animal CHF standard introduced by Mendel [38]. The results showed significantly higher levels of serum BNP in the CHF model group compared to the sham operation rats (*P* < 0.05). In contrast, the serum BNP concentration was reduced in the ASIV-treated rats in comparison to the CHF model rats (*P* < 0.05) (Figure 1(e)). In addition, we analyzed the expression of BNP and ANP in heart tissue. Compared to the sham operation group, the protein expression of BNP and ANP in the CHF model group was significantly increased (*P* < 0.05). Compared to the CHF model group, the protein expression of BNP and ANP in the CHF+low-dose and CHF+high-dose ASIV group rats was significantly decreased (*P* < 0.05) (Figures 1(f)–1(h)). These results indicate that ASIV attenuates cardiac hypertrophy and improves cardiac function.

### 3.2. ASIV Ameliorates Ventricular Hypertrophy

**Figure 2:**
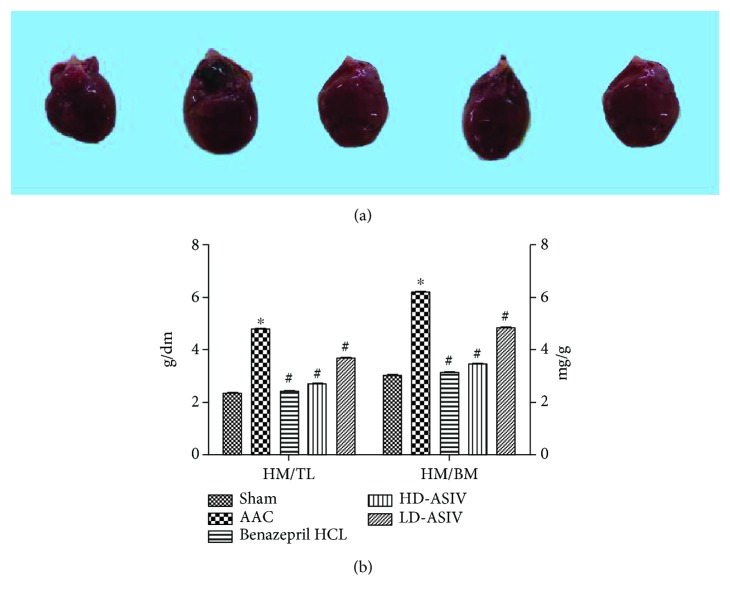
General parameters of ventricular hypertrophy. (a) Representative gross morphology of the whole heart. (b) Ratios of heart to body weight (HW : BW) and heart weight to tibia length (HW : TL) were used as a measure of cardiac hypertrophy. Quantitative data are shown as the means ± SD (*n* = 10 for the 5 experimental groups); ^∗^*P* < 0.05 vs. the sham operation group, ^#^*P* < 0.05 vs. the AAC group.

Rats in all groups were sacrificed painlessly, and their whole hearts were collected to measure their size. Compared to hearts from the sham operation group, the cardiac volume of the CHF model group was greatly enlarged, and the whole heart size was also increased (*P* < 0.05), as proven by HM/BM and HM/TL (Figures 2(a) and 2(b)). Compared to hearts from the CHF model group, the cardiac volume of the CHF+low-dose and CHF+high-dose ASIV group rats was much smaller, and the whole heart size was also decreased (*P* < 0.05).

### 3.3. ASIV Upregulates the Expression of Nrf2 and HO-1

**Figure 3:**
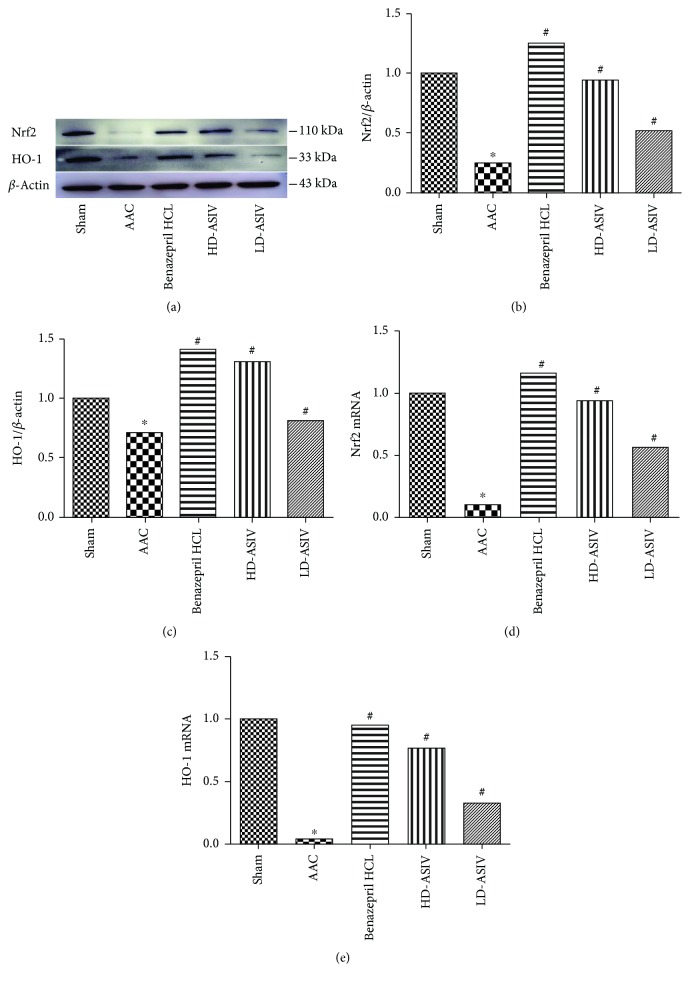
Western blotting and RT-qPCR analyses of antioxidative protein and mRNA expression in the 5 experimental groups. (a, b) Representative blots of Nrf2 and HO-1, and the intensity normalized against GAPDH, in the LV myocardium in the 5 groups (*n* = 10 for the 5 experimental groups). Quantitative data are presented as the means ± SD. ^∗^*P* < 0.05 vs. the sham operation group; ^#^*P* < 0.05 vs. the CHF model group. (c) Relative quantification of Nrf2 and HO-1 mRNA by RT-qPCR in the LV myocardium in the 5 groups (*n* = 10 for the 5 experimental groups). Quantitative data are presented as the means ± SD. ^∗^*P* < 0.05 vs. the sham operation; ^#^*P* < 0.05 vs. the CHF model.

To study the impact of ASIV on antioxidant stress, we analyzed the expression of Nrf2 and HO-1 (Figure 3). Compared to the sham operation group, the protein expression of Nrf2 and HO-1 in the CHF model group was significantly decreased (*P* < 0.05). Importantly, these effects were reversed in the CHF+low-dose and CHF+high-dose ASIV group rats (*P* < 0.05) (Figures 3(a)–3(c)). We also analyzed the mRNA levels of Nrf2 and HO-1 and found that their gene expression was significantly decreased in the CHF model group compared to the sham operation group (*P* < 0.05). Treatment with ASIV (in the CHF+low-dose and CHF+high-dose ASIV groups) overcame the inhibitory effects of CHF (Figures 3(d) and 3(e)). These results were consistent with the Western blotting data.

### 3.4. ASIV Prevents H9c2 Cardiomyocyte Hypertrophy

**Figure 4:**
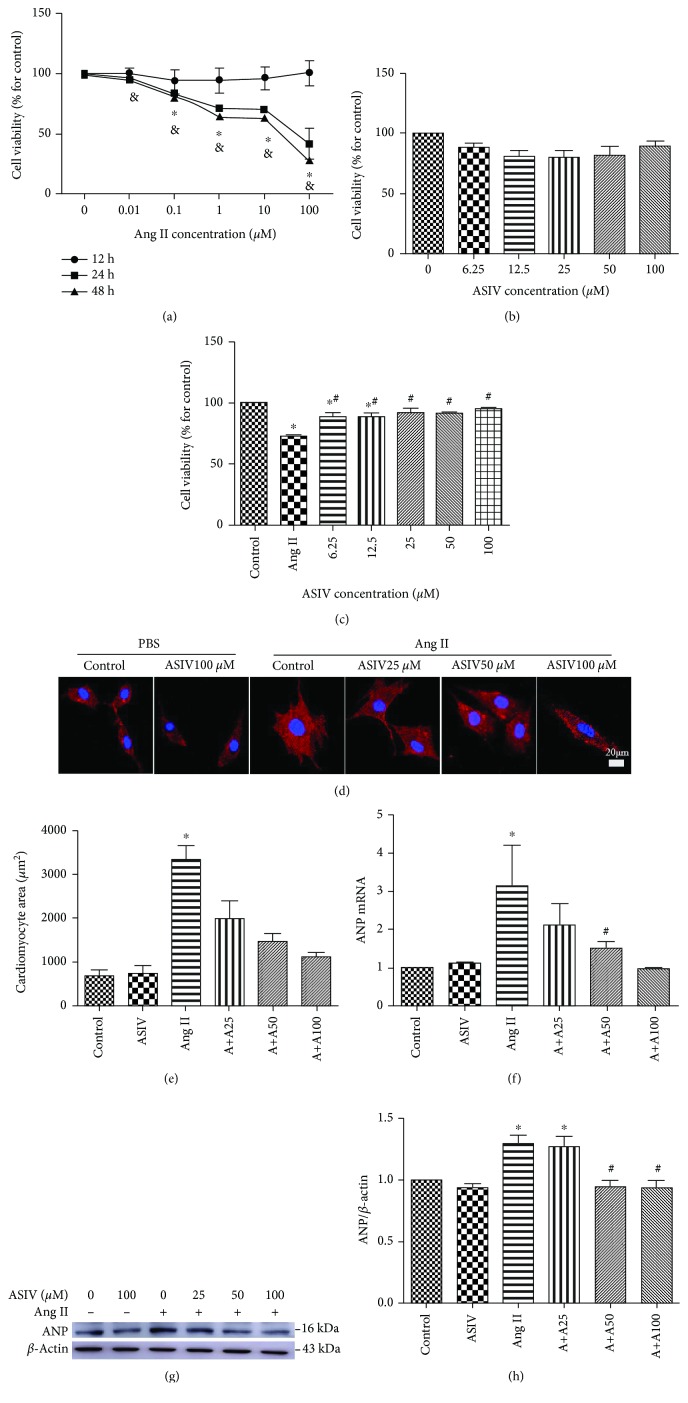
Effects of ASIV on the inhibition of H9c2 cell hypertrophy induced by Ang II. (a) To determine the optimal concentration and time of Ang II treatment, H9c2 cardiomyocytes were treated with 0, 0.01, 0.1, 1, 10, or 100 *μ*M of Ang II for 12, 24, or 48 h, followed by CCK8 analysis. (b) To investigate the cytotoxicity of ASIV, H9c2 cardiomyocytes were treated with 0, 6.25, 12.5, 25, 50, or 100 *μ*M of ASIV for 24 h, followed by CCK8 analysis. (c) After stimulation with 1 *μ*M of Ang II, H9c2 cardiomyocytes were treated with different concentrations of ASIV, and cell viability was measured with CCK8 assays. (d, e) Representative images and quantification of cardiomyocyte areas evaluated after *α*-actinin immunostaining. Scale bar, 20 *μ*m. *N* = 3. (f) The expression of a cardiac hypertrophic marker, atrial natriuretic peptide (ANP), at the mRNA level was detected by real-time fluorescence quantitative PCR. (g, h) The expression of ANP at the protein level was detected by Western blotting. ^∗&^*P* < 0.05 vs. the control group; ^#^*P* < 0.05 vs. the Ang II group.

The CCK8 assay showed that there was no significant difference in cell viability between the different Ang II (0, 0.01, 0.1, 1, 10, and 100 *μ*M) groups cultured for 12 h. However, a remarkable decrease in cell viability compared to the control group was observed between the different Ang II (0.1, 1, 10, and 100 *μ*M) groups cultured for 24 h or 48 h according to the CCK8 assay (Figure 4(a)). When H9c2 cardiomyocytes were treated with 0, 6.25, 12.5, 25, 50, or 100 *μ*M ASIV for 24 h, no significant differences were observed in the CCK8 assay (Figure 4(b)). Treatment with ASIV at concentrations of 6.25-100 *μ*M increased cell viability compared to the Ang II group (Figure 4(c)). The cellular surface areas of cardiomyocytes were obviously increased in the Ang II groups (Figures 4(d) and 4(e)**)**. ASIV (25, 50, or 100 *μ*M) pretreatment decreased Ang II-induced cardiomyocyte enlargement in a dose-dependent manner compared with the Ang II group, which was accompanied by significant decreases in the cardiac hypertrophy marker ANP (Figures 4(d)–4(h)).

### 3.5. ASIV Alleviates Oxidative Stress and Upregulates the Expression of Nrf2 in Enlarged Cardiomyocytes

**Figure 5:**
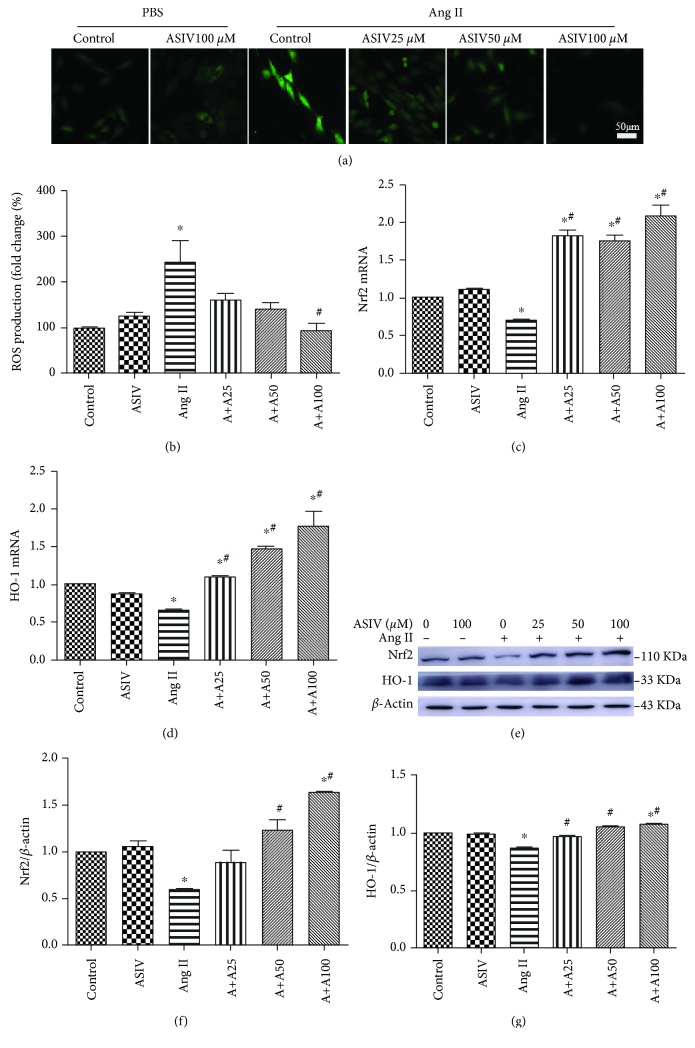
ASIV regulates the level of ROS generation and the expression of Nrf2 in hypertrophic cardiomyocytes induced by Ang II. (a, b) ROS generation was detected by DCFH-DA staining. Data are expressed as the mean ± standard deviation, *N* = 3. (c, d) Nrf2 and HO-1 expression at the mRNA level was detected by real-time fluorescence quantitative PCR. (e-g) Nrf2 and HO-1 expression at the protein level was detected by Western blotting. ^∗^*P* < 0.05 vs. the control group; ^#^*P* < 0.05 vs. the Ang II group. ROS: reactive oxygen species. DCFH-DA: 2 ′,7 ′-dichlorofluorescein diacetate.

The results described above indicated that ASIV could prevent Ang II-induced cardiomyocyte hypertrophy. In addition, ASIV pretreatment was able to alleviate Ang II-induced ROS production (Figures 5(a) and 5(b)). Therefore, whether ASIV prevents this Ang II-induced effect by activating Nrf2 was examined by measuring the Nrf2 expression in cardiomyocytes. The expression of Nrf2 and HO-1 at both the mRNA (Figures 5(c) and 5(d)) and protein (Figures 5(e)–5(g)) levels was significantly increased in the enlarged cardiomyocytes treated with ASIV (25, 50, or 100 *μ*M), and these changes appeared in a dose-dependent manner.

### 3.6. Knockdown of Nrf2 Expression by Plasmid Transfection

**Figure 6:**
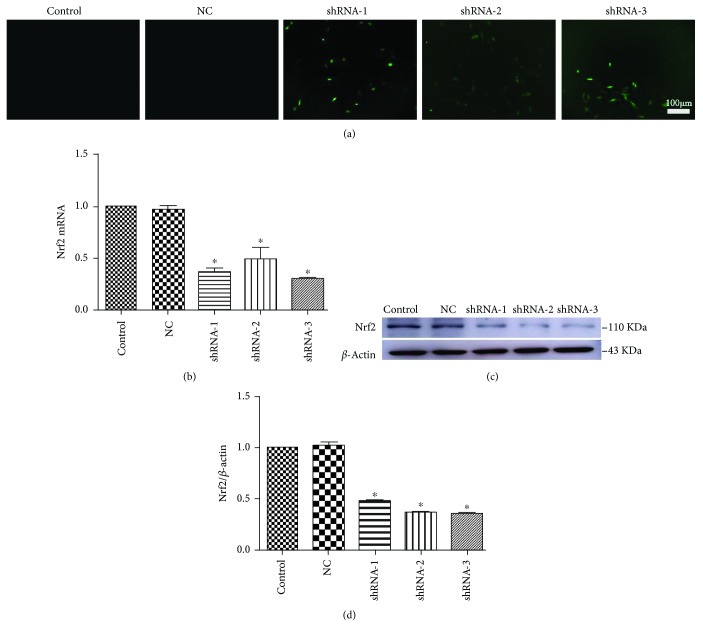
Knockdown of Nrf2 expression by plasmid transfection of Nrf2-shRNA in H9c2 cardiomyocytes. (a-d) H9c2 cardiomyocytes were transfected with Nrf2-negative control shRNA (NC) or different Nrf2-shRNA sequences (shRNA-1/shRNA-2/shRNA-3) and then cultured for 24-36 h. (a) The expression of green fluorescent protein in transfected H9c2 cardiomyocytes was observed by fluorescence microscopy. (b) The expression of Nrf2 at the mRNA levels was detected by real-time fluorescence quantitative PCR. (c, d) The expression of Nrf2 at the protein level was detected by Western blotting after transfection. ^∗^*P* < 0.05 vs. the control group.

To determine the effect of Nrf2-shRNA transfection, H9c2 cardiomyocytes were transfected with the Nrf2-negative control shRNA (NC) or different Nrf2-shRNA sequences (shRNA-1, shRNA-2, and shRNA-3). The expression of green fluorescent protein in transfected H9c2 cardiomyocytes was observed by fluorescence microscopy (Figure 6(a)). There was no significant difference in the expression of Nrf2 between the control and NC groups, whereas the expression of Nrf2 at both the mRNA (Figure 6(b)) and protein (Figures 6(c) and 6(d)) levels was significantly decreased in Nrf2-shRNA transfected H9c2 cardiomyocytes.

### 3.7. The Protective Effect of ASIV against Cardiomyocyte Hypertrophy Induced by Ang II Occurs through Stimulation of the Nrf2/HO-1 Pathway

**Figure 7:**
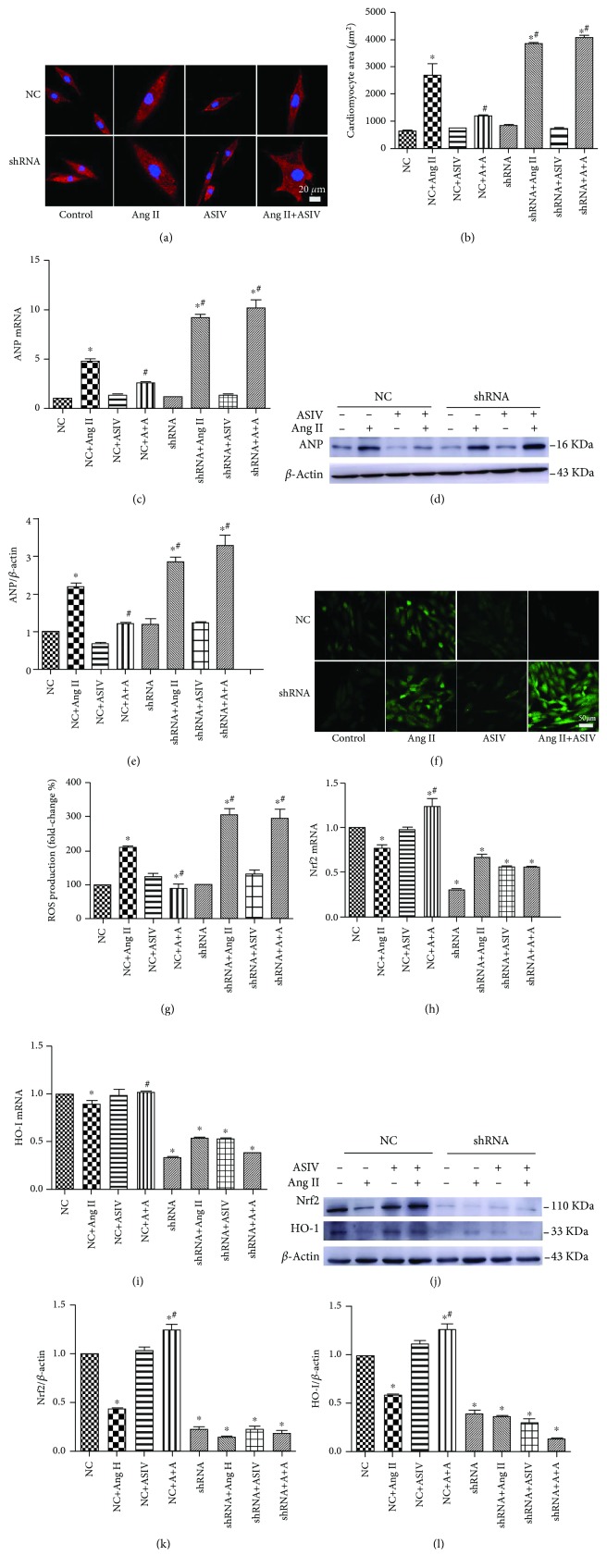
Knockdown of the Nrf2 gene abolishes the protective effect of ASIV against Ang II-induced cardiomyocyte hypertrophy. (a-f) H9c2 cardiomyocytes were transfected with Nrf2-negative control shRNA (NC) or Nrf2-shRNA-3 (shRNA) and then stimulated with Ang II (1 *μ*M) or ASIV (100 *μ*M) for 24 h. (a, b) Representative images and quantification of the cardiomyocyte area evaluated after *α*-actinin immunostaining. Scale bar, 20 *μ*m. *N* = 3. (c) The mRNA expression of ANP was detected by real-time fluorescence quantitative PCR. (d, e) The protein expression of ANP was detected by Western blotting in H9c2 cardiomyocytes. (f, g) ROS levels detected by DCFH-DA in the indicated groups (*n* = 3 samples per experimental group, magnification: 200x). (h, i) The mRNA expression of Nrf2 and HO-1 was detected by real-time fluorescence quantitative PCR. (j-l) The protein expression of Nrf2 and HO-1 was detected by Western blotting in H9c2 cardiomyocytes. ^∗^*P* < 0.05 vs. the control+NC group, ^#^*P* < 0.05 vs. the Ang II+NC group.

The direct role of Nrf2 in ASIV-mediated cardiomyocyte protection against Ang II was further confirmed via shRNA knockdown. After transfection with Nrf2 shRNA or negative control shRNA for 24 h, the cardiomyocytes were treated with Ang II (1 *μ*M) alone, ASIV alone (100 *μ*M), or the combination of Ang II (1 *μ*M) and ASIV (100 *μ*M) for an additional 24 h. In negative control cardiomyocytes, Ang II treatment caused significant cardiomyocyte hypertrophy, as indicated by an increased cell surface area and upregulation of the expression of ANP at both the mRNA and protein levels. All of these cardiomyocyte changes were prevented by ASIV treatment (Figures 7(a)–7(e)). Compared with negative control cardiomyocytes, however, the Nrf2-shRNA cardiomyocytes treated with Ang II showed further enlargement of the cell surface area and increased ANP expression. Additionally, ASIV treatment did not prevent Ang II-induced cardiomyocyte hypertrophy in Nrf2-shRNA cardiomyocytes (Figures 7(a)–7(e)).

ASIV reduced the increase in the ROS level induced by Ang II in negative control cardiomyocytes. In contrast, Nrf2 silencing enhanced the increase in the ROS level induced by Ang II, which could not be ameliorated by ASIV (Figures 7(f) and 7(g)). In Nrf2-shRNA cardiomyocytes, RT-qPCR and Western blot analyses revealed that the expression of Nrf2 was significantly decreased compared with the negative control, regardless of ASIV administration (Figures 7(h)–7(l)). The expression of the Nrf2 downstream gene HO-1 showed the same trend (Figures 7(h)–7(l)). These results indicated that silencing of the Nrf2 gene abolished the ASIV-mediated activation of antioxidant gene downstream of Nrf2.

## 4. Discussion

In this study, we proved that ASIV protected cardiac function and reversed cardiac hypertrophy in CHF rat models of AAC and hypertrophic cardiomyocytes induced by Ang II. We further demonstrated that ASIV is associated with the inhibition of cardiac hypertrophy by activating the Nrf2/HO-1 pathway. Therefore, this study provides direct evidence that administration of ASIV is a safe, effective strategy for preventing cardiac hypertrophy and improving cardiac function.

Previous studies have demonstrated the cardiovascular protective effect of ASIV from various perspectives. For example, ASIV has been shown to protect cardiomyocytes from hypoxia-induced injury, possibly via downregulation of miR-23a and miR-92a and activation of the PI3K/AKT and MAPK/ERK signaling pathways [14]. Wan et al. [39] found that the preventive effects of ASIV and its active sapogenin cycloastragenol on cardiac fibrosis in mice occurred by inhibiting the NLRP3 inflammasome. Another study indicated that ASIV might be a potential candidate for inhibiting apoptosis and inflammation, thereby preventing cardiac hypertrophy via elevating the suppressor of IKK*ε* (SIKE) to suppress TBK1/PI3K/AKT activity [40]. In addition, Tang et al. [37] reported that ASIV inhibited ventricular remodeling, improved cardiac function, and decreased the free fatty acid (FFA) concentration in CHF rats [36]. Our results are consistent with these studies, and we demonstrated that ASIV attenuates cardiac hypertrophy both in vivo and in vitro (Figures 1(a)–1(h), 2(a) and 2(b), 4(d)–4(h), and 7(a)–7(e)).

Oxidative stress indicates an imbalance between the production of ROS and the antioxidant buffering of ROS and/or nitrogen species. As second messengers and signal transduction regulators, ROS are a key trigger of cardiomyocyte hypertrophy and apoptosis [41]. Oxidative stress has been identified as one of the key determinants of the pathogenesis of heart disease, including atherosclerosis, diabetic cardiomyopathy, postmyocardial infarction remodeling, and heart failure [42, 43], and increased ROS levels are considered to be a major driver of pathology and tissue degeneration [44]. It has been established that the Ang II-mediated cardiac disease is mainly induced by oxidative stress. Ang II interacts with its receptor AT (mainly AT1) to induce ROS production mediated by nicotinamide adenine dinucleotide phosphate oxidase, which disrupts endogenous antioxidant defenses, leading to oxidative stress [45]. As we discovered, Ang II promotes cardiomyocyte hypertrophy (Figures 4(d)–4(h)) and induces the production of ROS (Figures 5(a) and 5(b)).

Nrf2 is a transcription factor involved in stress responses. It regulates the expression of antioxidant defense genes such as HO-1 and NQO1 by binding to ARE in the promoter region under internal and external stress conditions [46, 47]. The Nrf2 defense system is highly sensitive to changes in the cellular redox balance during the development of cardiovascular diseases and associated complications [48–51]. HO-1 is one of the most relevant targets of Nrf2 in endothelial homeostasis, which is usually parallel to the upregulation of ferritin, hence reducing free iron levels and preventing Fenton-type reactions. Signorelli et al. [52] indicated that plasma HO-1 is decreased in peripheral artery disease patients. Previous studies have shown that myricetin alleviates oxidative stress, inflammation, apoptosis, and fibrosis through upregulation of the Nrf2/HO-1 signaling pathway, thereby potentially protecting against diabetic cardiomyopathy [53]. Another study demonstrated that ginsenoside Rg1 exerts antioxidant stress, hence protecting cardiomyocytes against hypoxia/reoxygenation injury via activation of Nrf2/HO-1 signaling [54]. Tebay et al. [55] have proved that excessive intercellular ROS levels protect cardiomyocytes by inducing the target antioxidant gene of Nrf2 to activate Nrf2. In turn, activation of the Nrf2 defense system reduces oxidative stress injury by regulating ROS and inflammatory processes [56]. ROS stimulate the activity of antioxidant proteins such as Nrf2, which can be used as a negative feedback mechanism [57]. As an antioxidant, it has been proven that ASIV inhibits ROS production and activates the Nrf2 signaling pathway in neuronal cells [58]. The current study revealed that ASIV significantly attenuated cardiac hypertrophy in CHF rat models of AAC and hypertrophic cardiomyocytes induced by Ang II via activating the Nrf2/HO-1 pathway (Figures 3(a)–3(e), 5(c)–5(g), and 7(h)–7(l)). Furthermore, we showed that ASIV alone could not increase the expression of Nrf2 but only increased Nrf2 expression when cardiomyocytes were stimulated by Ang II to activate the oxidative stress system. ASIV, as an antioxidant, has been shown to inhibit ROS generation via activation of Nrf2/HO-1 signaling to protect against cardiomyocyte hypertrophy.

## 5. Conclusions

**Figure 8:**
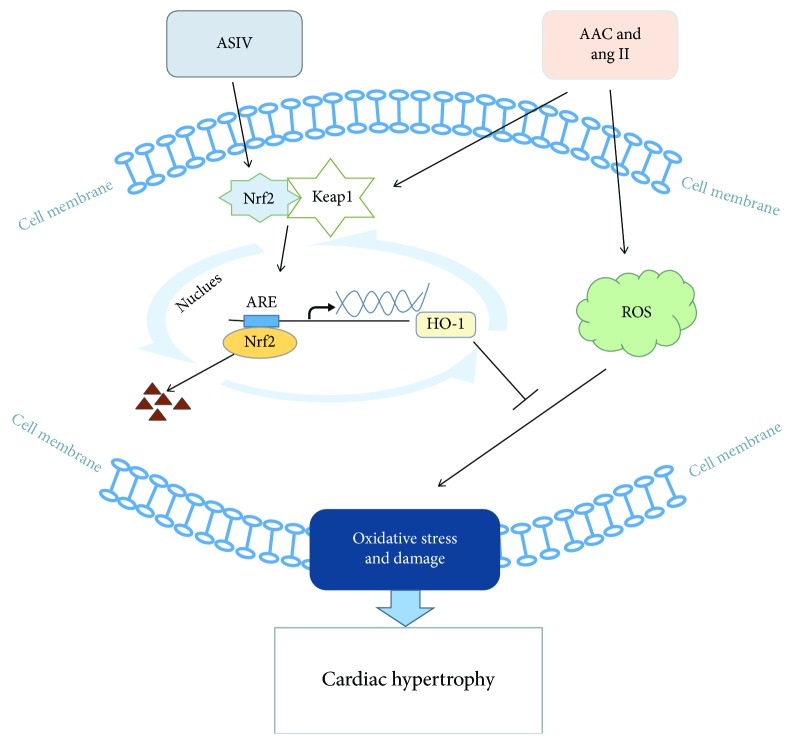
Schematic diagram depicting the effects of ASIV on cardiac hypertrophy via activating the Nrf2/HO-1 signaling pathway in vivo and in vitro. Stimulation due to AAC surgery and Ang II-induced ROS production. ASIV intervention activated Nrf2 and promoted the expression of the downstream antioxidant gene HO-1, inhibiting the production of ROS and, thus, ameliorating oxidative stress and damage and, ultimately, cardiac hypertrophy.

In conclusion, our study clearly highlights that ASIV inhibits cardiac hypertrophy partially by activating the Nrf2/HO-1 pathway (Figure 8). This work provides novel insights into the mechanisms underlying ASIV-induced protection against cardiac hypertrophy and provides strong evidence of the mechanism whereby ASIV attenuates cardiac hypertrophy and improves cardiac function. However, in addition to regulating oxidative stress, ASIV can also play a role in cardioprotective effects related to other aspects, and its specific mechanisms need to be further explored. Furthermore, in clinical treatment, traditional Chinese medicine mostly involves the preparation of the ASIV compound as a monomer, and its extraction and purification still need to be further improved. In future research, further efforts in the above areas will be required to provide a new direction for the clinical treatment of cardiovascular diseases.

## Data Availability

The data used to support the findings of this study are available from the corresponding author upon request.
